# Social gradient in the cost of oral pain and related dental service utilisation among South African adults

**DOI:** 10.1186/s12903-016-0313-x

**Published:** 2016-11-05

**Authors:** Imade J. Ayo-Yusuf, Sudeshni Naidoo

**Affiliations:** 1 Department of Dental Management Sciences, School of Dentistry, Faculty of Health Sciences, University of Pretoria, PO Box 1266, Pretoria, 0001 South Africa; 2 Department of Community Oral Health, Faculty of Dentistry, University of the Western Cape, Private Bag X1, Tygerberg, 7505 South Africa

**Keywords:** Oral pain, Cost of oral pain, Social gradient, Socio-economic position, South Africa, Access to care

## Abstract

**Background:**

Oral pain affects people’s daily activities and quality of life. The burden of oral pain may vary across socio-economic positions. Currently, little is known about the social gradient in the cost of oral pain among South Africans. This study therefore assessed the social gradient in the cost of oral pain and the related dental service utilisation pattern among South African adults.

**Methods:**

Data were obtained from a nationally representative cross-sectional survey of South African adults ≥16 year-old (*n* = 2651) as part of the South African Social Attitudes Survey conducted by the South African Human Sciences Research Council. The survey included demographic data, individual-level socio-economic position (SEP), self-reported oral health status, past six months’ oral pain experience and cost. The area-level SEP was obtained from the 2010 General Household Survey (*n* = 25,653 households) and the 2010/2011Quarterly Labour Force Survey conducted in South Africa. The composite indices used for individual-level SEP (α = 0.76) and area-level SEP (α = 0.88) were divided into tertiles. Data analysis was done using t-tests and ANOVA. Significance was set at *p* < 0.05.

**Results:**

The prevalence of oral pain among the adult South Africans was 19.4 % (95 % CI = 17.2–21.9). The most commonly reported form of oral pain was ‘toothache’ (78.9 %). The majority of the wealthiest participants sought care from private dental clinics (64.7 %), or from public dental clinics (19.7 %), while the poorest tended to visit a public dental clinic (45 %) or nurse/general medical practitioner (17.4 %). In the poorest areas, 21 % responded to pain by ‘doing nothing’. The individual expenditure for oral pain showed a social gradient from an average of ZAR61.44 spent by those of lowest SEP to ZAR433.83 by the wealthiest (national average ZAR170.92). Average time lost from school/work was two days over the six-month period, but days lost was highest for those living in middle class neighbourhoods (3.41), while those from the richest neighbourhood had lost significantly fewer days from oral pain (0.64).

**Conclusions:**

There is a significant social gradient in the burden of oral pain. Improved access to dental care, possibly through carefully planned universal National Health Insurance (NHI), may reduce oral health disparities in South Africa.

## Background

Oral pain may have an impact on the daily activities of sufferers, as it affects their quality of life. The most common type of oral pain is toothache [1, 2]. The experience of toothache is more common among people of lower socio-economic position [3–10]. This phenomenon can be attributed, firstly, to the higher prevalence of oral disease among the poor [11] and, secondly, to delays in seeking dental treatment [10]. The presence of oral pain may not override or negate the barriers experienced by people of low socio-economic position who wish to access a dental facility, but the severity of the pain that people experience appears to be associated with the timing of the poor’s seeking dental care [10]. When they are faced with such barriers, some poor people may seek alternative ways to alleviate their pain. Some consult traditional healers to relieve toothache – in a study from one developing country, as many as 60 % of those who experienced toothache reported seeking treatment from traditional healers [12]. The cost of care could be an important factor preventing the poor from seeking care [13].

The cost of oral pain can be considered in three dimensions. One measure for the cost of oral pain is the direct financial cost of the condition to the individual. The other measure is the indirect cost, which is associated with loss of productivity such as the days lost from work. This includes cost of lost wages to the individual and cost associated with loss of productivity to the work place; this has an impact on the national economy with a potentially large cumulative cost [14]. The indirect cost also includes time lost for activities such as studying time, or students’ absence from school or university. The third measure is the intangible cost which relates to the suffering experienced by individuals with oral pain, this dimension affects the quality of life of the individual.

Some of the literature suggests that areas in which people live may influence their health status. In particular, the socio-economic position of the area has been shown to influence the health status of the population [15, 16]. However, the effect of a person’s area-level socio-economic position on the cost of oral pain and the individual’s response to oral pain in South Africa is not yet fully understood.

Understanding the cost of oral pain, especially in relation to the areas in which people live, could assist in oral health services planning. This is particularly important at this time, since South Africa is now planning for the implementation of a National Health Insurance (NHI).

The aim of this study was to assess the direct (individual financial cost) and indirect cost (time lost from work/school) of oral pain and related dental service utilisation patterns among different socio-economic groups in the South African adult population.

## Methods

### Study design and sample population

This study used three cross-sectional nationally representative datasets. All these datasets involved a multi-stage cluster sampling technique using the same master sample. The primary survey was part of the Human Sciences Research Council’s (HSRC) annual South African Social Attitudes Survey (SASAS) (*n* = 3003; 85 % response rate) for 2011. This is an interviewer-administered household survey carried out between September and November 2011 on a representative sample of South African adults aged 16 years and older. The sample was drawn from the master sample of the HSRC, which consisted of 1050 enumeration areas (EAs) drawn from the 2001 national census. From each of the EAs, 10 visiting points were randomly selected giving rise to a total of 10,500 visiting points in the master sample. Stratification of the EAs was done by the socio-demographic domains of province, geographical sub-types, tribal areas (formal rural, formal and informal urban) and the four population groups (Statistics South Africa, 2001). However, for the SASAS, 3500 households/visiting points were randomly selected from the master sample. Each person was then randomly selected from each household without replacement. Efforts were made to secure interview with selected person by making 3 visits before registering the person as non-responding.

No specific sample size was determined for this study as it was a secondary data analysis and predetermined master sample for the primary dataset. However, post-hoc power analysis showed that the study was sufficiently powered for detecting socio-economic position group differences in the prevalence and cost of oral pain and related dental service utilisation.

This survey used a structured questionnaire to obtain information on individual-level socio-demographic characteristics including highest educational attainment, current employment status, income, household assets owned, subjective socio-economic position, possession of a private health insurance (medical aid) and the oral health related data (past 6 months prevalence and cost of oral pain; and the service utilisation pattern associated in response to the last oral pain episode). The questionnaire used in this survey was first pilot tested (to test questions included in the study for clarity and comprehension) prior to data collection.

The prevalence of oral pain over the past six months was obtained by asking the participants the question “In the past 6 months have you had any pain from the following in your mouth/jaw – teeth, gums, denture, sores around the mouth, jaw joint?” The response options were “No” or “Yes”.

The cost of oral pain was calculated as the direct financial cost for the last oral pain episode determined in South African Rand (ZAR), and indirect cost which was determined in terms of the number of days lost from work or school due to oral pain. For the indirect cost, the participants were asked the open-ended question “How many days in total did you miss from work, college or school in the past six months due to oral pain and/or a visit to the dental clinic?” The intangible cost of oral pain in this study was determined in terms of pain and suffering regarding access or lack thereof and the utilisation of dental services in response to oral pain experienced. The service utilisation patterns in response to oral pain was obtained by asking the respondents the question “What did you do/where did you go for your last pain episode with your teeth, dentures or mouth?” Options were given and multiple responses were allowed. The response options to this question included “Never had a painful dental episode before”, “Visited a private dental clinic”, “Visited a government dental clinic”, “Visited a nurse/GP/Hospital”, “Used self-medication/Pharmacist”, “Used home remedies”, and “Did nothing”.

The last dental visit payment method was obtained by asking “How did you pay for your last visit to the dental clinic?” only one response was requested. The options given were categorised as follows: Free (This included the response “I did not pay, the treatment was free”); Medical aid (This included the options “I paid the complete cost through my medical aid”, “I paid part through my medical aid and part from my pocket”, and “I paid with cash to collect a refund from my medical aid later”); and Cash (This included those who indicated “I paid with cash”). Those who did not visit a dental clinic were regarded as missing values, for the purpose of this sub-analysis focused on those who made a dental visit.

Two secondary datasets were used to ascertain respondents’ area-level socio-economic position. The General Household Survey (GHS) 2010 (*n* = 25,653 households; response rate 93.4 %) [17] and the four rounds of Quarterly Labour Force Survey (QLFS) (second, third and fourth quarters of 2010 and the first quarter of 2011 ~ 30,000/Quarter) were used [18–21]. Both surveys were conducted by Statistics South Africa, based on census enumeration areas similar to those for the primary dataset. The primary and secondary datasets were matched, based on the census enumeration areas.

Considering that socio-economic position is a multi-dimensional construct, a composite index was used as the measure of socio-economic position at both the individual level and the area level. This was used because it has the advantage of describing more fully the overall nature of socio-economic position compared to the use of a single variable.

#### Individual-level socio-economic position

The individual-level socio-economic position index was determined by first selecting the socio-economic variables that have been theoretically and/or empirically associated with health.

Each of the selected variables had different variances. As a result, the variables were standardised using the z-scores before carrying out a principal component analysis (varimax rotation) and a reliability test of the composite index. The individual-level socio-economic position index was determined after the principal component analysis (varimax rotation) of the variables, using a cut-off extraction factor of 0.4 for inclusion in the index.

The three final variables included in the index for individual-level socio-economic position were the asset index composed of a number of assets, formal educational attainment (number of formal schooling years, categorised as ‘<12 years’, ‘12 years’ and ‘> 12 years’ of formal education), and subjective socio-economic position (the position in which each participant would place him/herself in society on a scale of 1 to 10, with 10 indicating the top of society and 1 indicating the bottom of society).

##### Asset index

The asset index, a composite index, was derived from participants’ household asset ownership. The asset index (Cronbach alpha = 0.91) is a summed index score of the best fitting items obtained from a principal component analysis of a participant’s household assets. These household items include an electric stove, TV set, washing machine, microwave oven, DSTV, home theatre system, fixed Telkom phone line, radio, DVD player, vacuum cleaner, fridge, hot running water, computer and car.

Employment status did not meet the cut-off criteria, so it was excluded from the final index. Income was not included, because many data were missing for this variable, this observation is consistent with the published literature [22].

The final three items used were extracted as one component and explained 65.6 % of the total variance in the sampled population, with an excellent internal consistency or reliability (Cronbach alpha = 0.74).

#### Area-level socio-economic position

The area-level socio-economic position was measured using an index determined by means of principal component analysis (varimax rotation) of a set of selected variables obtained from the secondary datasets. Since the variances within each variable were different, each variable was weighted using its z-score to give equal weights prior to the principal component analysis. The variables used in the final index because the outcome of their factor analysis was favourable were the following:

- educational attainment (the percentage of people who had formal education of 12 years – ‘high school’/matriculation or more in the area),
- economic activity (the percentage of people who were eligible for employment and who were actually employed; and the percentage of people employed in the formal sector in the area),
- infrastructure (the percentage of homes in the area with flushing toilets and percentage of home in the area with piped water), and
- access to private health facilities (the percentage of people in the area who used private facilities when ill).

The six items used extracted as one component and explained 63.8 % of the total variance in the sampled population. This area-level socio-economic position index produced excellent internal consistency or reliability (Cronbach alpha = 0.88).

Both the individual-level and area-level socio-economic position index scores were auto-ranked and the total study population was divided into tertiles, namely lowest third, the middle third and the highest third. The lowest tertile of the corresponding index was used to represent the low socio-economic position for the individual and area levels respectively.

### Ethics

This study received ethical clearance from the University of the Western Cape Senate Research Ethics Committee [Reference number: 11/1/48] and from the Human Sciences Research Council (HSRC) Research Ethics Committee [Reference number: 5/17/08/11].

### Data analysis

All data analyses were conducted using SPSS version 22.0. All the analyses were weighted. Data analysis included t-tests for independent samples and ANOVA. All the statistical analyses were two-tailed. The threshold for statistical significance was set at *p* < 0.05.

## Results

The past 6-month oral pain prevalence among South African adults was 19.4 % (Table 1). Toothache was the most commonly reported type of oral pain (78.9 %). The prevalence of oral pain shows a significant social gradient by area-level with those living in the richest areas reporting the lowest oral pain prevalence. Wealthier people were significantly more likely to visit a private dental clinic in response to their last pain episode, and less likely to visit a government/public dental clinic. People who sought care from a general medical practitioner/nurse/hospital were significantly more likely to be in the lowest socio-economic position category. Those respondents who claimed that they ‘did nothing’ in response to their last pain episode were significantly more likely to be found in the poorest areas (Table 1).

**Table 1:** Prevalence of oral pain and type of health service sought in response to the last pain experienced by area-level and individual-level socio-economic position (SEP)

	Area-SEP % (95 % CI) n				Individual-SEP % (95 % CI) n			
Response	Low	Middle	High	*p*-value	Low	Middle	High	*p*-value
Oral pain prevalence	20.2 (16.5–24.3) (*n* = 185)	23.9 (19.6–28.9) (*n* = 209)	14.4 (11.3–18.0) (*n* = 122)	<0.05	21.7 (18.0–25.9) (*n* = 191)	19.1 (15.7–23.2) (*n* = 167)	16.9 (13.7–20.7) (*n* = 158)	
Private dental clinic	20.8 (12.7–32.2) (*n* = 44)	35.0 (27.0–43.9) (*n* = 81)	42.0 (30.0–55.0) (*n* = 46)		11.9 (7.5–18.5) (*n* = 23)	28.3 (19.3–39.3) (*n* = 45)	64.7 (53.4–74.5) (*n* = 103)	≤0.005
Public dental clinic	36.4 (27.0–47.0) (*n* = 70)	43.6 (33.8–53.9) (*n* = 78)	30.7 (21.3–42.1) (*n* = 45)		45.0 (36.0–54.4) (*n* = 90)	42.6 (32.5–53.4) (*n* = 72)	19.7 (12.0–30.6) (*n* = 31)	≤0.005
Nurse/GP/hospital	11.9 (6.4–21.2) (*n* = 23)	14.7 (8.0–25.7) (*n* = 25)	3.4 (1.5–7.7) (*n* = 8)		17.4 (10.3–27.8) (*n* = 31)	6.7 (3.5–12.4) (*n* = 15)	6.2 (2.9–12.5) (*n* = 10)	≤0.005
Self-medication	7.8 (4.4–13.5) (*n* = 14)	5.7 (3.2–10.2) (*n* = 20)	12.3 (4.9–27.4) (*n* = 10)		10.0 (5.0–19.0) (*n* = 14)	8.1 (4.2–15.0) (*n* = 14)	5.1 (2.9–9.0) (*n* = 16)	
Home remedy	11.3 (6.8–18.1) (*n* = 24)	10.0 (6.1–15.9) (*n* = 28)	10.7 (6.0–18.2) (*n* = 15)		11.1 (6.7–18.0) (*n* = 28)	12.9 (8.1–19.9) (*n* = 27)	7.0 (3.4–13.9) (*n* = 12)	
Did nothing	20.8 (13.2–31.2) (*n* = 26)	2.5 (0.7–8.3)^*^ (*n* = 3)	4.7 (1.9–10.9)^a^ (*n* = 8)	<0.005	13.3 (8.0–21.3) (*n* = 21)	9.8 (4.4–20.4) (*n* = 10)	6.4 (2.4–15.6) (*n* = 6)	

^a^Unreliable estimates due to small numbers; *p-value <0.005

A significant association was found between the payment method for the last dental visit and different socio-economic groups, both at the individual level and the area level (Table 2). Private health insurance (medical aid) was the predominant method of payment for those in the highest socio-economic position and those who live in the wealthiest areas. People in other areas were more likely to pay for their last dental visit with cash (Table 2).

**Table 2:** The payment method for the last dental visit by socio-economic position (SEP)

Measure		Free % (95 % CI) n	Medical aid % (95 % CI) n	Cash % (95 % CI) n	*p*- value
Individual SEP	Low	53.7 (44.5–62.6) (*n* = 164)	11.0 (5.3–21.4)^a^ (*n* = 25)	35.3 (27.1–44.5) (*n* = 91)	<0.001
	Middle	30.9 (24.7–38.0) (*n* = 126)	22.4 (16.5–29.6) (*n* = 71)	46.7 (39.0–54.6) (*n* = 149)	
	High	6.8 (4.5–10.1) (*n* = 34)	61.2 (54.6–67.3) (*n* = 287)	32.1 (26.5–38.2) (*n* = 172)	
Area SEP	Low	32.5 (25.1–40.8) (*n* = 102)	21.9 (15.5–30.0) (*n* = 71)	45.7 (36.7–54.9) (*n* = 112)	0.001
	Middle	28.1 (21.6–35.8) (*n* = 117)	34.5 (26.5–43.6) (*n* = 155)	37.5 (30.1–45.2) (*n* = 165)	
	High	22.9 (17.2–29.8) (*n* = 105)	46.5 (39.2–53.9) (*n* = 157)	30.7 (24.4–37.8) (*n* = 135)	

^a^Relative Standard Error >30 %, thus estimate here may be unreliable

**Fig. 1:**
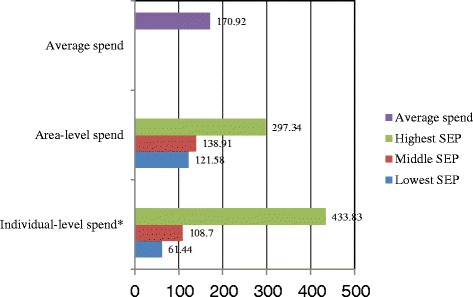
Financial cost (in South African Rand - ZAR) for the last oral pain episode by socio-economic position

The average amount spent on the last pain episode differed significantly across the different individual socio-economic groups. The national average amount spent on the last pain episode was R 170.92 (SE 33.14) (Fig. 1). If this national average amount spent on the last oral pain episode is multiplied by the total count of those people who experienced oral pain, the direct cost translates to an estimated total amount spent of R 991 118 052 (95 % CI: 532 656 996–1 549 802 324) over the six-month period prior to this study for the country.

The indirect cost measured by the number of days lost from work or school revealed a national average of 2.04 days lost from work or school per person who experienced oral pain within the six-month period (Table 3). The cost of days lost i.e., indirect costs, could not be reliably measured in financial terms as the income data of the participants was incomplete due to a lot of missing data on personal and household income.

**Table 3:** Days lost from work or school as a result of last oral pain episode by socio-economic position

		Mean number of days lost	Standard error	95 % Confidence interval	*p*-value
Individual SEP	Low (*n* = 191)	2.79	1.15	0.53–5.04	0.563
	Middle (*n* = 167)	1.52	0.62	0.31–2.73	
	High (*n* = 158)	1.51	0.37	0.79–2.23	
Area SEP	Low (*n* = 185)	1.63	0.69	0.28–2.97	0.037
	Middle (*n* = 209)	3.41	1.21	1.02–5.80	
	High (*n* = 122)	0.64	0.20	0.24–1.04	
South Africa	National (*n* = 516)	2.04	0.53	1.00–3.09	

## Discussion

This study’s finding suggests one in five South Africans had experienced oral pain in the last six months. While there was no significant variation in the prevalence of oral pain across socio-economic position at individual level, there was a significant social gradient in oral pain prevalence at area level, with oral pain prevalence being lowest in the richest neighbourhoods. Furthermore, just over half (57.2 %) of those in the areas in the poorest tertile responded to their last dental pain episode by attending a dental clinic, irrespective of whether it was a private or a public (government) dental clinic. This proportion of people is relatively low, compared to those residing in the middle tertile (78.6 %) and the richest (72.7 %) areas (Table 1). This suggests socio-economic disparities in the burden of oral health in terms of the prevalence of oral pain and access to dental care across the different areas. A few possible suggestions for this finding may be proposed, for example, a lack of accessible dental clinics in the poorest areas, in terms of geographic reach and/or affordability. Furthermore, people in poor areas may be unable to afford a dental visit, as about a third of people in the poorest areas claimed to have paid with cash for their last dental visit. There may be competing financial needs of higher priority than visiting a dental clinic to alleviate pain. They may therefore have developed tolerance to the pain as a coping strategy, given their economic/financial burden [23]. Lack of access to affordable dental care in the poorest areas may also explain why a fifth of the adults ‘did nothing’ in response to their last oral pain experience. Another reason for the relatively low dental service utilisation in poor areas could be that people do not know what to do about oral pain (a low dental IQ).

On average, the direct individual cost to treat the last oral pain episode within the past six months was ZAR170.92. A statistically significant direct financial cost gradient was observed across the SEP categories, demonstrating that the amount paid increased progressively from those in the poorest group to those in the wealthiest tertile. However, this difference could also be a reflection of the type of service(s) sought and/or received. Wealthier people were more likely to go to private clinics and were more likely to pay using private health insurance (medical aid). The possession and use of private health insurance is likely to remove the financial burden for the cost of treatment from such people if they opt for a more conservative treatment in response to their dental problem – a conservative dental treatment option such as endodontic treatment is likely to be more expensive than extraction. Poor people who visit public (government) clinics for free dental treatment may not have much choice, as most of these clinics are overcrowded and overburdened [24] resulting in a higher likelihood of extraction as the treatment offered, instead of a conservative dental treatment option where this option would be possible. Those who attend private clinics and pay out-of-pocket with cash may opt for a treatment option that is affordable to them, and would therefore differ from individual to individual, depending on the person’s individual socio-economic position. This view is consistent with the observation that while the cost of care for the last pain episode did not vary significantly by area-level socio-economic position, there was significant variation by individual-level socio-economic position (Fig. 1).

Furthermore, if about one third of those in the lowest tertile socio-economic position paid out-of-pocket for their dental care, this group of less affluent people would have borne a disproportionate part of the total estimated amount spend of about ZAR1 billion spent on oral pain over the six-month period under review or of a possible ZAR2 billion over a year. This financial cost is a significant financial burden for caring for oral pain among South African adults of 16 years and older, which excludes indirect costs such as lost wages, and the cost to the economy for loss of productivity. This cost is high at a national level, considering that government expenditure on health in 2011 for South Africa totalled ZAR 121.9 billion, of which only ZAR 42.3 billion were spent on the district health services [25]. Given the many competing health challenges in the South African population, the cost of oral pain needs to be considered carefully when any universal plan for healthcare services is made.

The mean number of days lost from work or school as a result of oral pain in the past six months was two days, which could translate to about four days lost per person per annum. This estimated figure of two days lost per person per six-month period is much greater than the figure of 1.48 h lost per person per year reported by Gift et al. [14]. Therefore, it is likely to have a greater implication for the economy (in the case of work days lost) and academic achievement (in the case of school days lost). A possible explanation for the greater number of days lost in the present study compared to that reported by Gift et al. [14] is that in South Africa, as in other similarly lower-resourced settings, it could take a whole day to get through the queues to access public health services. The treatment recovery may also take longer, as treatment is more likely to be extraction, an invasive or surgical intervention [24]. Extraction is more likely to be chosen as a treatment option for most people because people often delay seeking treatment until a more advanced level of oral disease is experienced. Given the large number of people seeking care in public clinics with limited staffing levels, treatment options may also be limited. Furthermore, poor people who pay cash for treatment, perhaps in private clinics, are also likely to opt for the cheapest treatment, which may be extraction.

### Limitations of the study

Information on other costs associated with relief of oral pain, such as the cost of transportation and of medication, were not obtained. This study is subject to recall bias on the part of the respondents and to social desirability/acquiescence bias. The respondents were not asked what type of treatment they received for the last oral pain episode, as different treatment options could have resulted in the wide variation in cost for the visit, as observed in the different individual socio-economic groups. As a result the authors can only speculate on the reason for the differences in the cost of treatment in the different group.

## Conclusions

The findings of this study suggest that the physical and financial burden of oral pain is high and that there is socio-economic disparity in access to dental care particularly across the different areas. It is important to consider the cost of dental care (and a lack of such care) in planning affordable and sustainable national health insurance. Moreover, the indirect cost of two days lost from work/school per person who experienced oral pain in the six-month period under review needs to be addressed in order to reduce the cost to the economy.

## Abbreviations

GHS: General Household Survey
GP: General Practitioner (Medical)
HSRC: Human Sciences Research Council
NHI: National Health Insurance
QLFS: Quarterly Labour Force Survey
SASAS: South African Social Attitudes Survey
SEP: Socio-economic position
ZAR: South African Rand
